# From Serotonin to Neuroplasticity: Evolvement of Theories for Major Depressive Disorder

**DOI:** 10.3389/fncel.2017.00305

**Published:** 2017-09-28

**Authors:** Bangshan Liu, Jin Liu, Mi Wang, Yan Zhang, Lingjiang Li

**Affiliations:** Key Laboratory of Psychiatry and Mental Health of Hunan Province, Mental Health Institute, The Second Xiangya Hospital of Central South University, National Clinical Research Center for Mental Disorder, National Technology Institute of Psychiatry, Changsha, China

**Keywords:** major depressive disorder, serotonin, neuroplasticity, final common pathway, antidepressant efficacy

## Abstract

The serotonin (5-HT) hypothesis of depression has played an important role in the history of psychiatry, yet it has also been criticized for the delayed onset and inadequate efficacy of selective serotonin reuptake inhibitors (SSRIs). With evolvement of neuroscience, the neuroplasticity hypothesis of major depressive disorder (MDD) has been proposed and may provide a better framework for clarification the pathogenesis of MDD and antidepressant efficacy. In this article, we first summarized the evidence challenging the monoamine hypothesis and proposed that the antidepressant efficacy of SSRIs is not derived from elevated monoamine (5-HT, noradrenaline (NE), or dopamine (DA)) concentration or monoamine neurotransmission. Second, we reviewed the role of stress in the pathogenesis of MDD and gave a brief introduction to the neuroplasticity hypothesis of MDD. Third, we explored the possible mechanisms underlying the antidepressant efficacy of typical antidepressants in the context of neuroplasticity theory. Fourth, we tried to provide an explanatory framework for the significant difference in onset of efficacy between typical antidepressants and ketamine. Finally, we provided a brief summarization about this review article and some perspectives for future studies.

## Introduction

Major depressive disorder (MDD) is a highly prevalent and highly debilitating psychiatric disorder. MDD is the leading cause of disability worldwide with approximately 350 million people around the world suffering from this disorder, and the disease burden of depression has been considered to become the second highest among all diseases by 2020 (World Health Organization, 2016). However, despite the devastating burden of MDD, the pathogenesis of this complex disorder still remains unclear and the current available treatment for depression is also far from optimal (Collins et al., 2011). Specifically, clinical diagnosis of depression is still suffering from lack of objective diagnostic biomarker (Jentsch et al., 2015) and the overall remission rate of sequenced first-line antidepressant treatments (including drugs and cognitive behavioral therapy) for MDD is only about 60%–70% (Rush et al., 2006). Besides, the first-line drugs recommended for MDD in authentic MDD guidelines (most are selective serotonin reuptake inhibitors (SSRIs) and selective serotonin-noradrenaline reuptake inhibitors (SNRIs)) are often criticized by the delayed onset of efficacy, namely, it takes 2 weeks or longer on average for these drugs to work (Royal Australian and New Zealand College of Psychiatrists Clinical Practice Guidelines Team for Depression, 2004; National Institute for Health and Care Excellence, 2009; American Psychiatric Association Work Group on Major Depressive Disorder, 2010; Bauer et al., 2013; Kennedy et al., 2016).

The suboptimal clinical practice of MDD calls for deep understanding of the pathogenesis of MDD and development of more potent and fast-acting antidepressants. Although several hypotheses have been proposed for MDD, the monoamine (serotonin (5-HT), noradrenaline (NE) and dopamine (DA)) hypothesis is still the most prevailing hypothesis of MDD since most of the currently available antidepressants work on monoamine transporters or receptors. This hypothesis, initially based on the unintentional findings that chemical compounds inhibiting reuptake (imipramine) or metabolism (iproniazid) of monoamine neurotransmitters (5-HT and NE) would demonstrate antidepressant efficacy (Hirschfeld, 2000; Mulinari, 2012), claims that MDD is derived from deficiency of 5-HT and/or NE in the synaptic cleft, and antidepressant efficacy would be achieved by increasing 5-HT and/or NE in synaptic cleft through inhibiting clearance or promoting synthesis and release of these monoamines.

The monoamine hypothesis satiates the intense needs of interpretation for the mechanism of pathogenesis of MDD from academy, pharmacies and public population and has guided the development of new antidepressants in 1980s–2000s. Nevertheless, accounting the complicated and heterogeneous clinical manifestations of MDD to deficiency of a molecule is too simplistic and may misguide our understanding of the complexity of this disorder. Indeed as expected, numerous findings inconsistent with this hypothesis have arisen from daily clinical observations, clinical researches and preclinical studies since the proposal of this hypothesis, among which the most prominent findings are the delayed onset of efficacy and inadequate response/remission rate of typical antidepressants as illustrated above. These findings challenged the monoamine hypothesis on one hand, and promoted the evolvement of theories about depression on the other hand. Specifically, to make up for the shortage of monoamine hypothesis, researchers have proposed monoaminergic receptor hypothesis, signaling hypothesis, neuroplasticity hypothesis, etc. (Racagni and Popoli, 2008). These hypotheses evolved towards a more comprehensive and reasonable understanding of MDD and antidepressant efficacy, and the succeeding hypothesis may be totally different from the initial monoamine hypotheses.

## Increased Synaptic Serotonin (or NE, DA) Concentration Does Not Account for The Antidepressant Efficacy of Antidepressants

**Figure 1:**
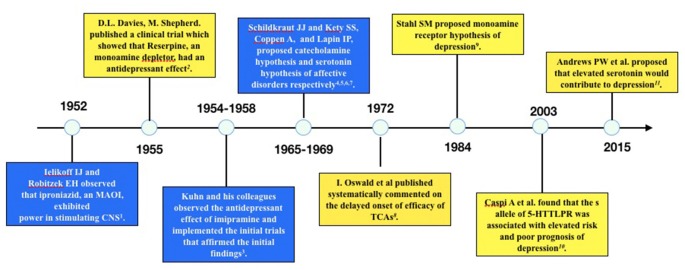
Timeline of historical events or publications supporting or opposing the monoamine hypothesis of depression. The blue boxes are events or publications supporting monoamine hypothesis and the yellow boxes are those opposing monoamine hypothesis. The following are the publications: 1. Selikoff et al. (1952), 2. Davies and Shepherd (1955), 3. Kuhn (1958), 4. Schildkraut (1965), 5. Coppen (1967), 6. Schildkraut and Kety (1967), 7. Lapin and Oxenkrug (1969), 8. Oswald et al. (1972), 9. Stahl (1984), 10. Caspi et al. (2003), 11. Andrews et al. (2015).

Several published reviews have casted doubt on the low 5-HT hypothesis of MDD and summarized the evidence inconsistent with this hypothesis (Lacasse and Leo, 2005; Racagni and Popoli, 2008; Fischer et al., 2014; Andrews et al., 2015). One article even hypothesized that depression is a result of elevated 5-HT concentration rather than deficiency of 5-HT (Andrews et al., 2015). The evidence challenging the low 5-HT hypothesis may be summarized as the following three categories: first, the rapid increase of 5-HT concentration in the synaptic cleft of neurons is inconsistent with the clinical delayed onset of antidepressant efficacy; second, lowering the concentration of 5-HT in synaptic cleft through tryptophan depletion (Ruhé et al., 2007) or serotonin transporter (SERT) enhancer (i.e., Tinaptine; Kasper and McEwen, 2008) failed to induce depression in healthy subjects, actually long-term antidepressants treatment had been detected to downregulating the total 5-HT concentration in the brain (Marsteller et al., 2007; Bosker et al., 2010; Siesser et al., 2013), which was contrary to the common sense of low 5-HT in depression; and third, genetic variants associated with potentiated SERT function (*l* allele of 5-HTTLPR) have been repeatedly found to be related with reduced risk of depression or better prognosis than variants associated with decreased SERT function (*s* allele of 5-HTTLPR; Karg et al., 2011). A timeline of historical publications or events supporting or opposing the monoamine hypothesis is shown in Figure 1.

The above findings together put sand in the wheels of low 5-HT hypothesis and indicate that it may not be reasonable to account the antidepressant efficacy of SSRIs to elevated 5-HT concentration or increased 5-HT neurotransmission in the brain. Thus the presumption that depression is caused by deficiency of 5-HT is also lack of solid basis. Actually, as stated in the *Stahl’s Essential Psychopharmacology: Neuroscientific Basis and Practical Applications*, “there is no clear and convincing evidence that monoamine deficiency accounts for depression, i.e., there is no “real” monoamine deficit” (Stahl, 2013). Similar opinions or comments from other authentic researchers or publications had been summarized in the impressive article of Lacasse and Leo (2005). Therefore, the low 5-HT hypothesis, although intriguing, are too simplistic and arbitrary for interpretation of the mechanisms underlying the complex manifestations of MDD.

To address the delayed onset of antidepressant efficacy, scientists further proposed the monoamine receptor hypothesis, which asserts that downregulation or desensitization of somatodendritic monoamine autoreceptor (such as 5-HT_1A_), rather than the elevation of monoamine concentration itself, is the key mechanism of antidepressant efficacy (Stahl, 2013). Since the somatodendritic 5-HT_1A_ autoreceptor inhibits impulse flow of 5-HT neurons, the downregulation or desensitization of this somatodendritic receptor induced by elevated concentration of 5-HT resulted from antidepressant intake would turn on neuronal impulse flow and bring about increased 5-HT in axonal terminals. The enhanced axonal 5-HT transmission and its subsequent neurobiochemical events, like regulation of gene transcription and protein synthesis, are deemed as the final mediators of antidepressant efficacy. As it takes several days to 2 weeks for the downregulation of 5-HT_1A_ autoreceptor to happen, the monoamine receptor hypothesis perfectly explained the delayed onset of antidepressant efficacy. However, both the clinical molecular imaging and postmortem studies failed to find consistent evidence supporting alterations of 5-HT_1A_ in patients with MDD (Ruhé et al., 2014). Besides, 5-HT_1A_ antagonists also failed to achieve consistent antidepressant efficacy in clinical trials. These research findings all casted doubts on the monoamine receptor hypothesis and calls for better hypothesis for the pathogenesis of depression.

Considering the antidepressant efficacy of electroconvulsive therapy (ECT), repetitive transcranial magnetic stimulation (rTMS), transcranial direct-current stimulation (tDCS) and new antidepressant ketamine and its derivatives, a legitimate inference might be that these therapies, although differed in forms and styles, would work on a final common pathway which underlies the pathogenesis of or vulnerability to MDD, and the antidepressant efficacy of these therapies is found on reversing or repairing the alteration of this final common pathway. Since no direct evidence about the association between 5-HT and depression and indirect evidence is highly inconsistent, there is no reason to claim that deficiency of 5-HT may serve as the “final common pathway” of depression. Then what else mechanism would be competent for the “final common pathway” of these diverse therapies? As has been repeated confirmed by preclinical and clinical studies, the relationship between stress and depression is robust and steady-going (Biegler, 2008; Risch et al., 2009; Binder and Nemeroff, 2010; Young and Korszun, 2010; Pizzagalli, 2014), thus it is legitimate to deduce that revealing the neurobiological sequelae of stress on the brain and its association with depression might provide insight in exploring the “final common pathway” of depression and antidepressant efficacy. Here we would like to take a brief look at the effect of stress on the brain and its role in the pathogenesis of depression at first.

## The Role of Stress in The Pathogenesis of MDD

In the framework of gene X environment for psychiatric disorders, stress is the validated environmental factor accounting to increased risk of development, exacerbation, chronicity and relapse of MDD. Generally, major depressive episodes (MDEs) are associated with about 2.5 times more frequent stressful life events in the period before episode as compared with comparable time period in controls (Hammen, 2005), and one stressful life event would lead to about 1.41-fold increased risk of MDE (Risch et al., 2009). In addition, stress is suggested to be linked with treatment resistance (Amital et al., 2008), poorer prognosis (Gilman et al., 2013) and higher rate of relapse and recurrence (Monroe and Harkness, 2005; Harkness et al., 2014) of MDD.

How should stress and depression be linked? Numerous theories has been proposed for interpretation of this phenomenon, among which the vicious circle between the dysregulation of hypothalamic-pituitary-adrenocortical (HPA) axis and morphological and functional deficits of hippocampal formation is considered as the key route between stress and depression. Specifically, the elevation of circulating cortisol during chronic stress response would exert neurotoxic effect on hippocampal neurons through glucocorticoid receptor and its downstream effects, which would result in decreased neurogenesis, synaptogenesis and dendritic spines and increased apoptosis of neurons (Holsboer and Barden, 1996; Holsboer, 2000; de Kloet et al., 2005). The morphological loss of neurons further leads to functional deficits loss of long-term potentiation (LTP) or long-term depression (LTD) of hippocampus, which gives rise to decreased GABAergic control of the HPA axis from the bed nucleus of stria terminalis (BNST) normally driven by the action of hippocampus (Holsboer, 2000; Egeland et al., 2015), and the the disinhibition of HPA axis would inversely exacerbate the morphological and functional loss of hippocampus. Thus, a vicious circle is formed and the hippocampal formation gradually goes to structural atrophy and functional deficit, which are commonly seen in depression.

Apart from the hypercortisolemia and deficits of hippocampal formation, the effect of stress on the biochemical metabolism and neurotransmission is also deemed to partly mediate the link between stress and depression. Biochemically, chronic stress would induce increased release of glutamate (Sanacora et al., 2012) in the hippocampus and prefrontal cortex (PFC), and blunted neurotransmission of 5-HT (Mahar et al., 2014) and DA (Pizzagalli, 2014) in mesocortical monoaminergic circuits. Specifically, chronic stress would downregulate the firing rate of dorsal raphe (DR) 5-HT neurons projecting to PFC and 5-HT_1A_ receptor sensitivity in PFC, which may be mediated by hypercortisolemia (Mahar et al., 2014). Similarly, diminished basal DA neuron firing in striatum is also observed in rodents exposed to chronic mild stress (Bekris et al., 2005). And, elevated release of glutamate in PFC is repeatedly observed after chronic stress, which is deemed to exert neurotoxic efficacy on the PFC and hippocampus neurons (Sanacora et al., 2012). These neurochemical changes would together result in negative influence on neuroplasticity through blunted neurogenesis, disrupted synaptogenesis, diminished dendritic spines and reduced synaptic connections. Besides, stress would diminish the cell proliferation and promote apoptosis of glial cells (Rial et al., 2015), which is the primary cell responsible for clearance of glutamate in the brain and may be responsible for the atrophy of hippocampus in MDD (Duman, 2004).

The functional and morphological changes of the brain induced by hypercortisolemia resulted from chronic stress are roughly consistent with the neuroimaging findings of abnormalities in MDD, i.e., atrophy and hypofunction of hippocampus and PFC, and hypertrophy and hyperfunction of amygdala (Andrade and Rao, 2010). Interestingly, the alterations in different brain regions may underlie different symptoms of MDD. Specifically, structural and functional alterations in the PFC-amygdala/hippocampus circuit may underlie depressive emotions; abnormalities in the PFC-nucleus accumbens (NAc) circuit may serve as the neural substrate of anhedonia (Phillips et al., 2015); and alterations of medial and dorsolateral PFC may mediate the cognitive dysfunction of MDD (Thomas and Elliott, 2009).

With the accumulated evidence supporting the strong correlation between stress and depression, and findings revealing the efficacy of stress on brain in line with the abnormalities found in MDD, the term “stress-induced depression” or at least “stress-correlated depression” would seem reasonable. As the case stands, the most frequently used and research validated depression animal model is the chronic stress induced depression model (Czéh et al., 2016). Thus, exploring the pathogenesis of MDD in the framework of stress-induced depression may be reasonable and necessary for our comprehending of this complex and heterogeneous psychiatric disorder.

The routes through which stress exert neurobiological effect on the brain as discussed above are all correlated with the growth, maturation, apoptosis and function of neurons. These processes, usually conceptualized as “neuroplasticity”, are of key significance in the pathogenesis of MDD. Therefore, they may also be competent for the role of “final common pathway” of antidepressant efficacy achieved by diverse treatment strategies. Below, we will give a brief introduction to the main contents of the neuroplasticity hypothesis of depression and take typical antidepressants and ketamine as examples to illustrate how neuroplasticity would serve as the “final common pathway” of antidepressant efficacy.

## Neuroplasticity Hypothesis of Depression: Main Contents

Although proposed for a long time and has won a lot of attention in academy, there is still no validated definition about the term “neuroplasticity”. Generally, neuroplasticity refers to the ability of neural system to adapt itself to the internal and external stimuli and to respond adaptively to future stimuli (Cramer et al., 2011). The processes of neuroplasticity are complex and the underlying mechanisms have not yet been fully understood, while it is widely accepted that the “neuroplasticity” includes both morphological and functional adaptation. Generally, the morphological neuroplasticity usually refers to neurogenesis, synaptogenesis, dendritic length and branching, spine density etc (Cramer et al., 2011; Egeland et al., 2015) and the functional neuroplasticity includes at least four forms: homologous area adaptation, cross-modal reassignment, map expansion and compensatory masquerade (Grafman, 2000). Neuroplasticity is of key significance in brain’s adaptation to stress, and maladaptive neuroplasticity may underlie various psychiatric disorders, such as depression, post-traumatic stress disorder, etc. Usually, the neuroplasticity theory of depression is usually supported by evidence from three domains (Serafini, 2012): (1) decreased neuroplasticity in hippocampus and PFC in depressed patients; (2) decreased concentration of neurotrophic factors, such as brain-derived neurotrophic factor (BDNF), in subjects with depression; and (3) antidepressants would elevate the concentration of neurotrophic factors and improve the neuroplasticity in hippocampus and PFC.

In addition, what deserves to be mentioned is the role of “metaplasticity” (a term coined by Abraham and Bear, 1996, meaning “plasticity of neuroplasticity”) in explaining stress-induced neural plasticity. The “metaplasticity”, or “activity-dependent and persistent change in neuronal state that shapes the direction, duration or magnitude of future synaptic change (Abraham and Bear, 1996)” in another way of saying, includes some key functions like preparing synapses for plasticity and learning and regulating synaptic plasticity homeostatically (Hulme et al., 2013). These functions may be achieved through actions on NMDA and metabtropic glutamate receptors (mGluRs) or heterosynaptic metaplasticity mechanisms like synaptic tagging and capture (Abraham, 2008; Hulme et al., 2013). Metaplasticity is sensitive to environmental stimuli, like environment enrichment or stress and dysregulation of metaplasticity induced by chronic stress may contribute to induction of depression (Vose and Stanton, 2017). For a detailed description of mechanisms underlying metaplasticity and their clinical relevance, the impressive articles of Abraham and Bear (1996), Abraham (2008) and Hulme et al. (2013) may be valuable.

As discussed above, with the establishment of stress-induced depression conceptual framework and the key role of neuroplasticity as mediator between stress and depression, neuroplasticity theory would be an optimal choice for understanding the pathogenesis of depression and antidepressant efficacy. Since we have illustrated the role of stress in the pathogenesis of MDD and the changes of brain induced by stress hereinbefore, next we will discuss how the antidepressants work on neuroplasticity.

## How Typical Antidepressants Work on Neuroplasticity?

The possible mechanisms of typical antidepressants on neuroplasticity have been reviewed in several articles (Racagni and Popoli, 2008; Andrade and Rao, 2010; Serafini, 2012; Harmer and Cowen, 2013; Hayley and Litteljohn, 2013). Briefly, antidepressants may improve neuroplasticity through the following pathways.

First, antidepressants improve neuroplasticity through monoamine neurotransmitters’ stimulation of the postsynaptic monoamine receptors. These receptors are mostly G-protein coupled receptor (GPCR) and would initiate subsequent signaling after stimulation. Specifically, stimulation of these receptors would activate the adenylate cyclase (AC), which would catalyze the ATP to cyclic adenosine monophosphate (cAMP), and cAMP would further activate the cAMP-response element binding protein (CREB) through activation of protein kinase A (PKA; Carlezon et al., 2005). The transcription factor CREB is responsible for gene expression of many proteins involved in the neuroplasticity of hippocampus, such as BDNF, glutamate receptor unit 1 (GluR1), etc (Pittenger and Duman, 2008). Since the atrophy of hippocampus has been consistently found to play a key role in the vulnerability, chronicity, and treatment-resistance of MDD (MacQueen and Frodl, 2011), improving the neurogenesis of hippocampus through activation of postsynaptic monoamine receptors may effectively promote depression recovery. This pathway may be abbreviated as the “GPCR-cAMP” pathway. While the “GPCR-cAMP” pathway is commonly seen in other organs or tissues, it is not the major pathway regulating the function of CREB in the brain (Carlezon et al., 2005).

Second, antidepressant would regulate neuroplasticity through reducing release of presynaptic glutamate, especially the depolarization-evoked release of glutamate, in PFC (Bonanno et al., 2005). The possible molecular mechanism of antidepressants on the release of glutamate had been reviewed in the article of Sanacora et al. (2012). The reduced glutamate release may imply decreased neurotoxic efficacy and strengthened synaptogenesis, synaptic connections and neurogenesis. To be mentioned, chronic antidepressant would also prevent the stress-induced glutamate release, which may underlie the clinical prophylaxic efficacy of maintenance antidepressant treatment for relapse or recurrence of MDE.

Third, antidepressant may work on neuroplasticity through enhancing AMPA to NMDA throughput (Du et al., 2006). Antidepressants may binding to the glycine-binding site of NMDA receptor and inactivate this site (Paul and Skolnick, 2003). The inactivation of NMDA receptor activity would result in inhibition of eukaryotic elongation factor 2 (eEF2) and enhance the expression of BDNF through subsequent signaling (Monteggia et al., 2013). Besides, antidepressant would upregulate the expression of AMPA subunits GluR1 and potentiate the function of AMPA (Martinez-Turrillas et al., 2002). The depolarization of AMPA receptor would activate the voltage-dependent calcium channels (VDCCs) and induce influx of Ca^2+^ into cytoplasm, which would further trigger the exocytosis of BDNF. Then the extracellular BDNF would further stimulate its membrane receptor—TrkB and regulate gene expression and neuroplasticity through subsequent signaling (Yoshii and Constantine-Paton, 2010). Thus stimulation of AMPA and inactivation of NMDA would work synergistically to improve neuroplasticity in the brain.

Fourth, antidepressant may improve neuroplasticity directly through LTP-like process. It has been repeatedly revealed that hippocampal synaptic plasticity was suppressed by stress through diminished amount of LTP, while antidepressant would reverse the negative efficacy of stress and potentiate synaptogenesis and synaptic connectivity through inducing LTP-like processes (Popoli et al., 2002; Shakesby et al., 2002).

Last but not least, antidepressant may also improve neurogenesis in the hippocampus through activation of the 5-HT_1A_ receptor (Santarelli et al., 2003).

Despite the role of BDNF in promoting neuroplasticity and neurogenesis in the hippocampus and PFC and mediating the antidepressant efficacy as mentioned above, what needs special attention is that BDNF may also promote neuroplasticity and neurogenesis in the amygdala, ventral tegmental area (VTA) and NAc, which is assumed to provoke depressive-like behaviors or exacerbate depressive symptoms (Racagni and Popoli, 2008; Harmer and Cowen, 2013; Hayley and Litteljohn, 2013). Thus, the antidepressant efficacy is not totally opposite to the site-specific neurophysiological and neurochemical efficacy of stress on different brain regions, which inhibits neuroplasticity, induces atrophy in hippocampus and PFC and promotes maladaptive neuroplasticity and induces hypertrophy in amygdala. The hypertrophy and elevated activation of amygdala may underline the heightened risk of relapse in recurrent MDD.

## Delayed Efficacy of SSRI and Fast Responding Ketamine: Clinical Trial Findings and Possible Interpretations of Discrepancy in Onset

The rapid onset of antidepressant efficacy of ketamine and delayed onset of efficacy in SSRIs treatment is of special interest. A meta-analysis revealed that overall response rate of single dose ketamine after 24 h is about 52.6%, and this efficacy would last about 3 days and decreased gradually with 10.9% of response rate remained at the end of week two after injection (Newport et al., 2015). Repeated ketamine infusions are associated with a relatively higher overall response rate (70.8%), and the efficacy lasts about 18 days on average after the last ketamine injection (Murrough et al., 2013). Although the clinical application of ketamine for depression is limited by its potential of abuse, the significant difference in time of efficacy onset between ketamine and typical antidepressants is of special clinical significance, since rapid onset of efficacy is urgently needed for MDD patients, particularly for those with suicidal ideation. Clarification the mechanisms underlying the discrepancy of efficacy onset between the two genre drugs may be helpful for the development of new antidepressants with rapid onset of efficacy.

The possible mechanism of antidepressant efficacy of ketamine has been summarized in several reviews (Browne and Lucki, 2013; Zunszain et al., 2013; Kavalali and Monteggia, 2015; Scheuing et al., 2015), which all stated that the blockade of NMDA receptor and potentiation of AMPA receptor is of key significance in ketamine’s antidepressant efficacy. NMDA and AMPA are two ionotropic glutamate receptors distributed widely in the brain. Their physiological ligand, glutamate, is the only excitatory neurotransmitter and innervates the majority of neurons in the brain. Neurohistological studies found that 85% of the brain mass are composed of neocortex, and glutamate is the primary neurotransmitter of 80% neocortex neurons and 85% neocortex synapses (Douglas and Martin, 2007). It is not difficult to infer from the above data that glutamate neurons account for so high proportion of the whole brain neurons that some researchers believe that the brain is largely a “glutamatergic excitatory machine” and all brain functions, particularly cognition and emotion are “ultimately mediated by the changes in excitatory transmission (glutamate) and its counterbalance of the inhibitory component (GABA)” (Sanacora et al., 2012).

As discussed above, glutamate is closely related to neuroplasticity in the brain. Release of glutamate may induce rapid LTP and promote synaptogenesis and synaptoconnectomes. Blocking NMDA receptor and activating AMPA receptor may promote the expression of BDNF gene and promote neuroplasticity synergistically. Thus glutamate is the primary system regulating neuroplasticity in the brain. With these arguments, we believe that the fast onset of antidepressant efficacy of ketamine may be explained by the following two reasons: (1) ketamine acts directly on NMDA receptor and indirectly on AMPA receptor, while SSRIs mainly act on SERT and indirectly regulate efficacy of glutamate receptors; although activating the postsynaptic monoamine receptors also plays a role in the neuroplasticity, this pathway is much slower and weaker than direct working on ionotropic glutamate receptors, i.e., NMDA and AMPA, as discussed above; and (2) the glutamate neurons and neurotransmitters account for much higher proportion in number of neurons and synapses than 5-HT neurons (and other monoamine receptors), drugs work on the glutamate system would exert much greater efficacy on the brain than drugs work on the 5-HT system. Namely, ketamine takes a faster speed and shorter route to regulate neuroplasticity than SSRIs, and this is why the fast responding of ketamine and delayed onset of SSRIs would occur.

## Summary and Future Perspectives

The monoamine theory of depression originated from the interpretation of the phenomenon observed in clinical practice, and has served as the primary hypothesis of MDD for more than 50 years. The prosperity of low 5-HT hypothesis is contributed to multilateral force coming from public, academy, industry, history, etc, as illustrated in the wonderful article of Mulinari (2012). However, with new observations and research evidence constantly emerging, this simplistic hypothesis has been intensely challenged and modifications or even totally new hypothesis are needed. Although SSRIs are currently first-line antidepressants in psychiatry practice, new efficacious drugs with rapid onset of efficacy are emerging. And, in theory research area, a paradigm shift has occurred from monoamine hypothesis to glutamate and neuroplasticity theory, which provides a more mature interpretation framework for the complicated psychiatric disorder.

Neuroplasticity hypothesis of MDD evolves from the monoamine hypothesis and tries to address the problems of monoamine hypothesis. This theory starts from the key role of stress in the pathogenesis of MDD, and provides a reasonable framework for the interpretation of the relationship between stress, brain, depression and antidepressant efficacy. Although the molecular mechanisms underlying neuroplasticity are not fully clarified, this hypothesis provides the most promising framework for understanding the pathogenesis of depression and antidepressant efficacy. However, there are some major themes urgently needed for clarification in future studies.

First, the relationship between stress and MDD has been extensively explored, while gene also plays a key role in the pathogenesis of MDD, how the interaction between gene and stress work on neuroplasticity and its relationship with depression pathogenesis and antidepressant efficacy is of special interest for scientists and clinicians.

Second, more comprehensive and detailed understanding of the molecular mechanisms, particularly the interaction between the neurotransmitter receptors and their subsequent signaling pathways, underlying neuroplasticity, depression and antidepressant efficacy is needed. Targets in these signaling pathways may be of special value in new antidepressant development.

Third, the neuroplasticity theory is not exclusive for MDD, it may also account for the pathogenesis of other psychiatric disorders, such as schizophrenia and bipolar disorder. Thus an interesting question is how the alterations in neuroplasticity account for the significantly different symptomatology of these disorders? Exploring answers to this question may help delineating the boundaries of MDD and searching for objective diagnostic biomarkers for MDD.

## Author Contributions

LL, YZ and BL co-designed the topic and contributed substantially to the conception of the article. BL performed the literature work, drafted the manuscript and approved the final version of the article. JL, MW and YZ contributed valuable suggestions to the conception of the article and partial literature analysis. LL and YZ critically revised the manuscript and have approved the final version of the article.

## Conflict of Interest Statement

The authors declare that the research was conducted in the absence of any commercial or financial relationships that could be construed as a potential conflict of interest.
